# pH‐Dependent Microenvironmental Ionic Signaling in Pancreatic Ductal Adenocarcinoma

**DOI:** 10.1111/apha.70183

**Published:** 2026-02-27

**Authors:** Albrecht Schwab, Micol Rugi, Pawel Swietach, Wiktoria Błaszczak, Ivana Novak, Ganga Deshar, Stine Falsig Pedersen, Renata Ialchina, Albin Sandelin, Jiayi Yao, Stephan J. Reshkin, Rosa A. Cardone, Tiago M. A. Carvalho, Annarosa Arcangeli, Rayhana Bouazzi, Franco N. D'Alessandro, Natalia Prevarskaya, Madelaine M. Audero, Halima Ouadid‐Ahidouch, Julie Schnipper, Luis A. Pardo, Xiaoyi Shi, Frauke Alves, Jakub Mitręga, Anna Trauzold, Sofie E. Hagelund, György Panyi, Marco Cozzolino, Clemens M. W. G. Löwik, Laura Mezzanotte, Roisin McMorrow, Andreas Pahl, Torsten Hechler, Elena Papacharisi, Alessandra Fiorio Pla, Ildiko Szabo, Verena Hofschröer, Zoltán Pethő

**Affiliations:** ^1^ Institut für Physiologie II University of Münster Münster Germany; ^2^ Department of Physiology, Anatomy and Genetics University of Oxford Oxford UK; ^3^ Section for Cell Biology and Physiology Department of Biology University of Copenhagen Copenhagen Denmark; ^4^ The Bioinformatics Center, Department of Biology University of Copenhagen Copenhagen Denmark; ^5^ Department of Biosciences, Biotechnology and Environment University of Bari Bari Italy; ^6^ Department of Experimental and Clinical Medicine, Section of Internal Medicine University of Florence Florence Italy; ^7^ Laboratory of Cellular Physiology (INSERM U1003) University of Lille Lille France; ^8^ Laboratory of Cellular and Molecular Physiology University of Picardie Jules Verne Amiens France; ^9^ Max Planck Institute for Multidisciplinary Sciences Göttingen Germany; ^10^ Institute for Experimental Cancer Research Christian‐Albrecht‐Universität Kiel Kiel Germany; ^11^ Department of Biophysics and Cell Biology, Faculty of Medicine University of Debrecen Debrecen Hungary; ^12^ Department of Radiology and Nuclear Medicine Erasums University Medical Center Rotterdam the Netherlands; ^13^ Heidelberg‐Pharma Research GmbH Ladenburg Germany; ^14^ Department of Life Sciences and Systems Biology, Department of Life Sciences and Systems Biology University of Torino Turin Italy; ^15^ Department of Biology University of Padua Padua Italy

## Abstract

**Aim:**

Pancreatic ductal adenocarcinoma (PDAC) develops within a uniquely dynamic pH landscape shaped by substantial acid–base fluxes produced by the exocrine pancreas. Secretion of alkaline pancreatic juice, normally linked to digestion, produces intermittent acidifications of the pancreatic interstitium, which challenges epithelial and stromal cells. It was postulated that these unique pancreatic pH dynamics can facilitate PDAC initiation and progression through selection of a more aggressive phenotype emerging with PDAC driver mutations.

**Methods:**

Here, we summarize evidence that pH‐regulatory transport proteins have an important role in shaping the PDAC microenvironment.

**Results:**

pH‐regulatory transport proteins generate and sense their microenvironment and act as signaling hubs to regulate proliferation, migration, and metabolism, and immune evasion. In this way, transport proteins that are crucial for the normal physiology of the exocrine pancreas are misused and become coerced into playing a pro‐cancer role in pancreatic tumor cells, pancreatic stellate cells, or infiltrating immune cells. Experiments with PDAC mouse models revealed a therapeutic potential of targeting pH dynamics, notably by inhibition or genetic ablation of pH‐regulatory proteins. It is a consistent finding that these maneuvers have a marked impact on the tumor immune defense and the communication between cancer and immune cells.

**Conclusion:**

Collectively, we present a case for considering pH‐regulating proteins as a therapeutic avenue.

## 1 Introduction

Pancreatic ductal adenocarcinoma (PDAC) is one of the cancers with the most detrimental prognosis with an overall 5‐year relative survival rate of only 13%. It is the third most frequent cause of cancer‐related death globally [1]. It arises in the exocrine pancreas that is unique in its ability to generate vast transepithelial acid–base fluxes, resulting in a characteristic pH landscape. Triggered shortly before and after each meal, the transport of HCO_3_^−^ into the lumen of pancreatic ducts is accompanied by a stoichiometric acidification of the pancreatic interstitium. Since the ductal HCO_3_^−^ concentration can reach 140 mmol/L, a marked albeit transient interstitial acidification is inevitable with each meal [2, 3]. The exact extent of the interstitial acidification is, however, challenging to verify experimentally; hence, its patho‐physiological role remains unclear. Yet, previous studies that showed interstitial acidification upon hormonal stimulation of HCO_3_^−^ secretion are proof‐of‐principle for this concept [4, 5].

In 2017, our consortium proposed that the intermittently acidic interstitium of the pancreas and the challenge this poses to intracellular pH (pH_i_) create a preconditioning niche that—in conjunction with specific driver mutations—can accelerate the progression of pancreatic cancer [3]. We assert that the physiological intermittent acidification prepares cells that reside in the pancreatic stroma for a faster course of disease progression, once PDAC has fully developed. The intermittent physiological acidification of the pancreatic stroma would then be overtaken by a more permanently acidic microenvironment [6], as is typically associated with solid tumors. The present review provides an overview of recent experimental evidence obtained in support of the idea that pH plays a decisive role in the pathophysiology of PDAC. Much of this work is the outcome of the European Marie Skłodowska‐Curie Innovative Training Network *pHioniC* (pH and Ion Transport in Pancreatic Cancer) on the role of pH and ion transport proteins in PDAC.

## 2 The Physico‐Chemical Signature of the PDAC Microenvironment

Pancreatic epithelial and stromal cells are exposed to a milieu of spatially and temporally varying extracellular pH (pH_e_), with episodes of substantial acidity and the emergence of transepithelial as well as interstitial pH gradients. Pancreatic ductal epithelial cells and the stromal cells in the surrounding interstitium are thus challenged to maintain pH_i_ constancy against a backdrop of large transcellular acid–base fluxes. The physiological acidification of the pancreatic interstitium is relevant for PDAC since it is well established that an acidic microenvironment drives cancer progression by selecting aggressive cellular phenotypes, for example, with higher metastatic potential including cancer stem cells [7, 8, 9, 10]. The genetic basis for pancreatic cancer cell aggressiveness lies most commonly in mutations in KRAS, TP53, SMAD4, and CDKN2A genes. PDAC is preceded by precursor lesions, PanIN1‐3, that can be dormant for decades and some may not develop into full‐blown disease [11]. At this stage, acid selection of specific cancer phenotypes may trigger disease progression. In this context, it is important to note that charge‐altering point mutations such as substitutions between histidine (with a side‐chain pK_a_ of 7.56) and arginine residues are among the most common mutations in cancers, including PDAC. By changing the protonation state of proteins (e.g., p53‐R273H and EGFR‐R776H), these mutations shift the pH optimum [12, 13]. Should these changes be selected positively, tumorigenicity increases. Recently, it was shown that the protonation of histidine residues within the DNA‐binding domains of transcription factors also confers a pH‐dependent regulation of transcriptional activity [14]. We therefore speculate that the unique pH dynamics in the normal pancreatic duct could facilitate progression toward invasive PDAC because tumor precursor cells are preconditioned by pH as a selection pressure. In this scenario, selection by pH synergizes with (charge‐altering) PDAC driver mutations, allowing cancer cells to proliferate beyond their normal restraints and checkpoints. These considerations are also relevant for other tumors since the above mentioned driver mutations are not PDAC‐specific.

In addition to its unique pH landscape, the PDAC microenvironment is characterized by distinct mechanical properties. Key features include an increased stiffness of the PDAC stroma and an elevated tissue pressure. The steady‐state modulus, a measure of tissue stiffness, of freshly dissected PDAC tissue is five times higher than that of healthy pancreatic tissue (5.46 ± 3.18 kPa vs. 1.06 ± 0.25 kPa) [15]. Pressure within the PDAC tissue reaches values of up to 100 mmHg so that blood vessels become compressed [16], causing hypoperfusion. The increased tissue pressure is largely attributable to the interstitial deposition of high‐molecular‐weight hyaluronan by pancreatic stellate cells that creates a large gel‐fluid phase [17, 18]. Moreover, the excessive production of extracellular matrix proteins, especially collagens, by pancreatic stellate cells and cancer‐associated fibroblasts (CAFs) contributes to tissue stiffness. Hypoperfusion of the PDAC tumor tissue, in turn, leads to the build‐up of acidic waste products, deprivation of metabolites and tumor hypoxia [19] and is among the causes of therapy failure because chemotherapeutic drugs cannot reach their intended targets [20].

## 3 Cells Within the PDAC Microenvironment

In the interest of space, we focus on the most prevalent cells in the PDAC microenvironment. Pancreatic stellate cells and cancer‐associated fibroblasts are important players in the PDAC microenvironment because they shape many of the physicochemical properties outlined above (see [21, 22, 23] for recent excellent reviews). These cells are responsible for the typical fibrosis (desmoplasia) of the PDAC tissue. The resulting increase in tissue pressure and stiffness, combined with the auto‐ and paracrine stimulation by the secretome of pancreatic stellate and cancer cells, respectively, results in a multilayered feed‐forward mechanism that perpetuates stellate cell activation and matrix secretion [24, 25, 26]. Stromal cells are also important in maintaining tumor cell metabolism in the hypoxic and nutrient‐deprived microenvironment by sharing key metabolites with tumor cells. Some examples include cancer‐associated fibroblasts providing amino acids such as alanine [27] or glutamine [28], lipids such as lysophosphatidylcholines [29] or nucleotides [30, 31] to cancer cells. Conversely, cancer‐associated fibroblasts benefit from lactate produced by pancreatic cancer cells [32]. We refer to recent reviews for a more detailed elaboration of this topic (e.g., [33]). Finally, lymphoid and myeloid immune cells are important constituents of the PDAC microenvironment. Based on the low abundance of CD3^+^ and CD8^+^ T cells infiltrating the tumor stroma, PDAC is categorized as a “cold” tumor. Together with evaluating the expression of anti‐programmed cell death ligand (PD‐L1), the detailed histological evaluation can be numerically expressed as an “immunoscore” which has strong predictive power with respect to overall survival (OS) and recurrence‐free survival (RFS) [34, 35].

## 4 Transport Proteins Modify, Sense, and Transduce Properties of the PDAC Microenvironment

**FIGURE 1:**
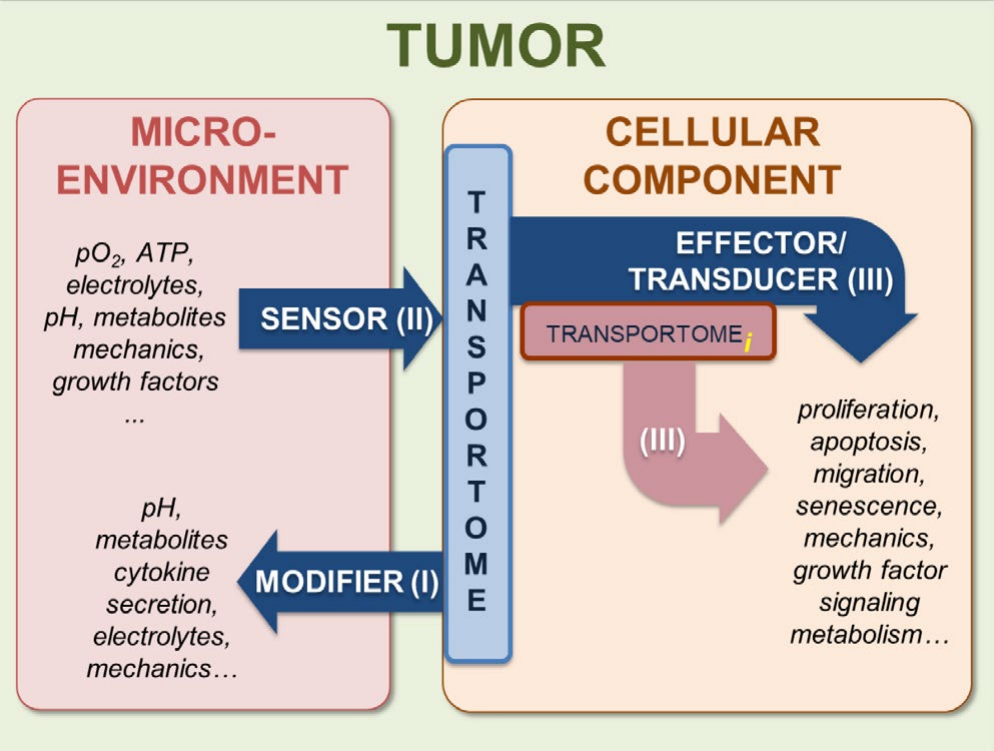
Schematic representation of the mode of action of transport proteins in PDAC pathophysiology. The transportome is either expressed in the plasma membrane or in the membrane of intracellular organelles (transportome_*i*_). In addition to being the modifier of the microenvironment, the transportome is also the acceptor of the signals from the microenvironment and part of signal transduction pathways in cancer and stromal cells.

The three fundamental principles pertaining to the PDAC transportome are summarized in Figure 1. These principles are not only valid for PDAC, but equally apply to other solid tumors and also to inflammatory diseases:

- Transport proteins *modify* the chemical properties of the PDAC microenvironment. For example, all cells of the PDAC tissue defend their pH_i_ homeostasis by exporting acid equivalents with the help of multiple transport proteins such as NHE1 [36], NBCs [37], MCT1, MCT4 [38, 39] or H^+^/K^+^‐ATPase [40, 41]. Because of the hypoperfusion of the PDAC tissue, acid accumulates in the tumor stroma [7]. Conversely, glucose is avidly taken up via GLUT1 [42] leading to its deprivation in the tumor stroma, whereas lactate is co‐exported with protons by the MCTs, leading to its accumulation in the stroma.
- Transport proteins *sense* the properties of the PDAC microenvironment. Interstitial acidity is, for example, *sensed* by various pH‐sensitive transport proteins that are not necessarily *bona fide* pH‐sensors like ASIC channels [43]. Yet, dynamic changes of the pancreatic pH landscape will inevitably lead to altered channel activity and downstream effects. Examples include specific K^+^ channels or Ca^2+^ permeable channels (see [44, 45] for review), ATP‐sensing purinergic P2X receptors [46, 47], or mechanosensitive channels such as Piezo1 [48] and K_2P_2.1 [49]. The extent to which transport protein‐mediated sensing is complemented by metabolic sensing of G‐protein coupled receptors, such as those for protons or lactate, is unclear.
- Finally, many transport proteins are central components of the signaling cascades underlying cancer and stroma cell behaviors such as proliferation [50, 51, 52], migration [50, 53], and apoptosis [54] etc. (see [55] for review). Thus, transport proteins *transduce* the cues received from the PDAC microenvironment [56, 57]. Numerous examples will be dealt with in the following sections of this review. Notably, the transport proteins transducing cues from the tumor microenvironment to cell behavior do not only reside in the plasma membrane but also in the membranes of intracellular organelles. Ion channels of the inner membrane of mitochondria stand out in this context. Their impact on (tumor) cell metabolism is an emerging branch of ion channel research in oncology [58, 59]. We envision that this field will rapidly grow in the near future.

## 5 Models and Methods to Mimic and Quantify the Pancreatic pH Landscape

Appropriate methods are needed to investigate the role of pH‐sensing/regulating transport proteins in PDAC pathophysiology. The seemingly simplest maneuver of setting the pH value of the CO_2_/HCO_3_^−^‐buffered culture medium to a desired value is often not performed with sufficient accuracy. For instance, in the case of media containing HCO_3_^−^, the pH value obtained by titration with HCl or NaOH “at the bench” in a nominally CO_2_‐free environment will be significantly more alkaline than that when such media are then returned to a CO_2_‐enriched atmosphere of incubators (see [60] for detailed protocols to set media pH correctly).

The duration of the experiments is another important aspect. Naming conventions such as “acute”, “short term”, “long term,” or “chronic” are often used, yet for correct interpretation it is necessary to indicate the exact duration of the experiments. When determining the pH sensitivity of a given transport protein itself, experiments with a time scale of several minutes are deemed necessary. This allows assessment of the activity of a given transport protein in a paired manner before and after a pH_e_‐ or pH_i_‐modifying maneuver (e.g., [61]). Significant changes in cellular behaviors are unlikely within such short time frames.

If a change in pH_e_ is maintained in vitro for several days to weeks, selection of a cellular phenotype with altered behavior will take place [62]. Since tumor acidification is typically a progressive process, a more accurate way of implementing pH changes is to periodically replace media with steps of lower pH. Acid‐adapted cells can also be used to model the metastatic escape of pancreatic cancer cells from the primary tumor by assessing their function after returning them to pH_e_ 7.4 [40, 63]. Both acid adaptation and acid selection will lead to the isolation of cells with markedly increased fitness to cope with the hostile acidic tumor microenvironment.

In an attempt to mirror the temporal evolution of the pancreatic pH landscape from the healthy pancreas to a fully developed PDAC, we cultured freshly isolated primary pancreatic stellate cells consecutively for 3 days each at pH_e_ 6.6, pH_e_ 7.4, and pH_e_ 6.6 [36]. Thereby, we sought to mimic the physiological (intermittent) acidification of the healthy pancreas, the lack of interstitial acidification and luminal HCO_3_^−^ secretion upon dedifferentiation of precursor lesions (PanINs) and the typical acidification of the tumor stroma, respectively. We are well aware that the latter model should be further refined in the future, for example, by the use of microfluidic devices that allow the application of temporal changes and spatial gradients of pH_e_ [64]. Thereby, the intermittent nature of the stromal acidification after each meal could be replicated experimentally.

So far it is not yet clear whether acid selection or adaptation prevails in the development of PDAC, as data on the evolution of the pancreatic pH landscape are missing. Adaptation processes are probably more important due to the gradual time course of PDAC development. Therefore, we propose to use acid‐selected or, preferably, acid‐adapted cells when studying the influence of pH on the behavior of tumor (stroma) cells. While mRNA and protein expression levels change significantly within 3 days, for example [36], a more complete adaptation to acidosis takes much longer, likely due to the selection of the “fittest” cells. For this reason, many studies have used growth in an acidic medium for a month or longer [62, 63, 65, 66, 67, 68, 69]. Finally, whenever possible, cells grown in a three‐dimensional (3D) environment, which better replicates the cancer microenvironment than standard 2D cell culture, should be preferred [70, 71, 72].

To the best of our knowledge, in vivo “pH‐targeting maneuvers”, that is, silencing or inhibiting pH‐regulatory (transport) proteins, have been explored only in very few studies using the numerous murine PDAC models [73]. Three studies used mice to investigate the roles of NBCe1 (*Slc4a4*), carbonic anhydrase IX and NHE1, respectively [36, 37, 74]. Another study showed that acid‐adapted pancreatic cancer cells are more metastatic when orthotopically transplanted into mice [75].

Numerous probes and methods are available to determine the intra‐ and extracellular pH ranging from pH‐sensitive microelectrodes [4] to fluorescent small molecules or genetically encoded pH indicators [76]. Attaching the small molecule indicators to a “targeting moiety”, for example, phosphoethanolamine, allows the quantification of the pericellular pH_e_ at the cell surface [77]. Alternatively, genetically encoded pH sensors like pHoran4 can be targeted to the extracellular side of the plasma membrane. This allows to record the impact of altered H^+^ transport on the pericellular pH_e_. Pericellular pH_e_ of PDAC cells rises in 2D and spheroid cultures when the H^+^/K^+^‐ATPase‐mediated H^+^ extrusion is inhibited with pantoprazole [40]. pH (low) insertion peptides (pHLIP) [78] were used to determine the acidic pH within the PDAC tumor microenvironment [6]. So far, targeted pH sensors have not yet been used to precisely map the pancreatic pH landscape during the evolution of PDAC. Targeting could be achieved genetically by promotor‐controlled organ‐specific expression or by the use of PDAC‐specific peptides as delivery agents [79]. Clearly, more studies employing advanced imaging sensors are needed to obtain detailed information on the dynamics of the interstitial and intracellular pancreatic pH landscape. When combined with a cell‐specific expression and crossbred with genetically engineered PDAC mouse models, such studies would deliver an unprecedented wealth of information on pH dynamics during the disease course of PDAC.

## 6 Acidity of the PDAC Microenvironment Shapes Tumor and Stromal Cell Behavior

### 6.1 Pancreatic Cancer Cells

The impact of the tumor microenvironmental acidosis on pancreatic cancer cells has been studied in several recent papers using the model of acid‐adapted pancreatic cancer cells or organoids. Transcriptomic analyses and functional studies indicate that acid adaptation leads to the appearance of a more aggressive phenotype, which is particularly evident when acid‐adapted PDAC cells are returned to normal pH_e_ to mimic invasive or metastatic cells escaping acidic regions [40, 62, 63, 67]. Hotspots of pH‐dependent transcriptional alterations include metabolism, extracellular matrix remodeling, cell cycle regulation, epithelial‐mesenchymal transition (EMT), DNA damage response, and cancer stemness [75]. The pattern induced by the acid adaptation in vitro correlated with that of pancreatic cancer tissue compared with non‐cancerous tissue. This clearly validates the in vitro cell model [80].

Functional studies revealed that acid‐adapted cancer cells have an increased net acid extrusion capacity, so that their pH_i_ at pH_e_ 7.4 is higher than in non‐adapted cells [81]. In addition, their pH_i_ regulation also depends on the composition of the extracellular matrix [65]. Acid adaptation increases growth in 3D culture, adhesion‐independent colony formation, and invasive outgrowth [63] as well as in vivo metastasis [75]. In addition, metabolism is markedly altered with a shift towards oxidative metabolism, fatty acid uptake, and increased peroxisome proliferator‐activated receptor‐α (PPARA, PPARα) activity [82, 83]. The analysis of different environmental stressors of pancreatic cancer cells revealed that the extracellular acidosis appears to be the dominant factor that shapes cancer cell metabolism when compared with hypoxia, glucose deprivation, and extracellular lactate accumulation. Unfortunately, medium pH was often set with the inaccurate “HCl titration method,” and pH_i_ was not considered in this study [84]. pH_i_, which is an important regulator of glycolytic enzymes, is lowered in parallel to an extracellular acidification: A one pH‐unit drop of pH_e_ is followed by a 0.6 pH‐unit decrease of pH_i_ in pancreatic cancer cells. Due to the pH sensitivity of glycolytic enzymes, lactate production is reduced when pH_e_—and hence pH_i_—is reduced [39]. A more detailed analysis of lactate handling in pancreatic cancer cells revealed that lactate can be shuttled between cancer cells via connexin‐43 channels from the hypoxic core of tumor cell spheroids to their normoxic periphery, where the concentration gradients for protons and lactate are permissive for MCT‐mediated cellular export [71]. Future studies will have to show whether dynamic changes of metabolic activity in pancreatic cancer cells [85] are related to the fluctuation of pH.

pH‐dependent phenotypic properties were linked among others to p53 signaling [63, 82] as well as to the expression and activity of transport proteins like NHE1 and Na^+^‐HCO_3_^−^ cotransporters (NBCs) [37, 81]. Depending on the use of cultured human cell lines or human and mouse tissue, different isoforms were described as the dominant ones: NBCn1 (SLC4A7) in cell culture [81], and NBCe1 (SLC4A4) in human and mouse tissue [37]. In addition to pH‐regulatory transport proteins, Ca^2+^ (permeable) channels like Orai [44] and TRPC1 [67] and pH‐sensitive K^+^ channels like K_2P_2.1 (TREK1) [40] contribute to the distinct phenotype of acid‐adapted or acid‐selected pancreatic cancer cells.

Therapy resistance is another important functional trait of acid‐adapted PDAC cells. Acid adaptation increases the expression of gemcitabine resistance genes in murine pancreatic cancer organoids [82] and the viability in the presence of chemotherapeutic drugs such as gemcitabine or erlotinib [65, 82]. Such a correlation between modulation of the pH of the PDAC microenvironment and response to gemcitabine was also inferred when additionally targeting carbonic anhydrase IX [74]. Combining gemcitabine with the CAIX inhibitor SLC‐0111 in different murine PDAC models (xenograft, orthotopic, PDX, genetically engineered Kras^G12D^/Pdx1‐Cre/p53/Rosa^YFP^) reduced tumor burden and prolonged animal survival. On a cellular level, CAIX inhibition reduced pH_i_ under hypoxic conditions and decreased gemcitabine‐induced glycolysis [74].

Acid‐adapted cells are more sensitive toward TRAIL, in particular when TRAIL is combined with an inhibitor of the anti‐apoptotic protein Bcl‐xL [66]. Notably, increased tumor cell killing can also be achieved by targeting TRAIL receptors and K_V_10.1 channels simultaneously by fusing an anti‐K_V_10.1 nanobody to TRAIL [86]. The modulation of cancer therapy failure by ion channels or transporters is an evolving theme in oncology. We refer to recent reviews for a detailed discussion of this emerging topic [87, 88, 89]. Many of the ion channels involved in therapy resistance of tumor cells, including those studied in PDAC such as K_2P_2.1 [40, 49, 52], TRPV6 [54] and Orai1 [90] are pH‐sensitive. To date, it remains elusive to which extent the therapeutic success of transportome targeting depends on the pH of the tumor microenvironment.

Modulators of channels or transporters can elicit their beneficial effects either by enhancing the sensitivity to chemotherapeutic drugs or by reversing mechanisms of resistance. Cancer cell stemness [75]—a known driver of resistance—is induced by acidosis so that it is a likely contributing mechanism. Modulation of tumor cell metabolism appears to be another important mechanism by which the pH homeostasis and/or transport proteins offer a new therapeutic window [59, 82, 83, 84]. It was suggested that lysosomal pH regulation can be exploited therapeutically by inducing an intracellular alkalinization, the so‐called alkaliptosis [91]. However, it is questioned whether the lysosomal channel TMEM175 acts as a proton channel in this process as suggested [92]. Moreover, pH_i_ measurements were made in the absence of HCO_3_^−^ which limits the ability of cells to counteract intracellular alkalinization.

### 6.2 Pancreatic Stellate Cells

The study of ion transport protein function in stellate cells is still in its infancy, yet several interesting findings have emerged. Studying the function of pancreatic stellate cells in vitro requires that they are exposed to an environment that differs drastically from their physiological one, in particular with respect to pH_e_ and mechanics. This has a major impact: It is a well‐known fact in the field that culturing primary pancreatic stellate cells under standard cell culture conditions inevitably leads to their activation. The activation of primary murine pancreatic stellate cells isolated from a healthy pancreas is prevented when they are cultured in an acidic medium (pH_e_6.6) [36]. Several mechanisms could account for a link between proton‐ and mechano‐signaling. The translocation of the transcription factor YAP‐1 to the nucleus of pancreatic stellate cells appears to be pH‐sensitive. It is found in the nucleus on a stiff substrate at pH_e_7.4 but not at pH_e_6.6 [36]. The RNAseq analysis showed that pH_e_ determines the phenotypic differentiation of primary pancreatic stellate cells: immunomodulatory at pH_e_6.6 and myofibroblastic at pH_e_7.4. The conclusion from this observation is that the intermittent acidification of the pancreas stroma after each meal can be seen as a “break” that maintains stellate cells in a quiescent state.

NHE1 is one of the major pH‐regulatory transporters in pancreatic stellate cells. Stellate cell activation is accompanied by a marked increase in NHE1 function, and it is in part NHE1‐dependent [36]. Once activated, pancreatic stellate cells produce excessive amounts of extracellular matrix causing the typical fibrosis of the tumor tissue. In vivo studies using the KPfC mouse model were in line with the in vitro data. Treating KPfC mice with the NHE1 blocker cariporide attenuates fibrosis of PDAC tissue which could at least in part be explained by a reduced activation of stellate cells and/or a reduced rate of proliferation [36]. An extracellular alkalinization following NHE1 inhibition could impair pericellular degradation of the extracellular matrix by pH‐sensitive matrix metalloproteases [93]. It is not yet known whether the antifibrotic effect of cariporide in KPfC mice is also due to reduced secretion of TGF‐β and other profibrotic cytokines as seen in lung fibroblasts [94]. Future studies have to test whether the decrease of fibrosis in the presence of cariporide involves a pH_i_‐dependent inhibition of Orai1 [95]. Orai1 activity promotes collagen secretion by pancreatic stellate cells [96] and its overactivation contributes to the progression of chronic pancreatitis, a disease associated with a marked fibrosis [97].

Electrogenic influx of Ca^2+^ ions into pancreatic stellate cells that can be mediated by Orai1 [96], TRPC6 [25], TRPV4 [61, 98, 99], TRPC1 [24, 100], or Piezo1 [48, 61, 98, 99], induces a depolarization of the cell membrane potential [101] which reduces the electrical driving force for further Ca^2+^ entry. In murine pancreatic stellate cells depolarization induced by Ca^2+^ influx is at least partially counteracted by the activity of K_2P_2.1 channels. This is particularly notable when pancreatic stellate cells are exposed to an elevated pressure in an acidic environment. Under these conditions the membrane potential of wildtype stellate cells is at ~−40 mV, while that of K_2P_2.1^−/−^ stellate cells is only at ~−20 mV. The differences in the cell membrane potential are accompanied by a failure of K_2P_2.1^−/−^ pancreatic stellate cells to adjust their migratory activity to the combined exposure of increased ambient pressure (+100 mmHg) and acidity (pH_e_6.6) [49]. Pancreatic stellate cells also express K_Ca_3.1 channels [53]. So far it is unknown whether these channels contribute to the pH‐dependent regulation of stellate cell behavior. While they are not mechanosensitive themselves, they could be indirectly activated following a mechanically induced Ca^2+^ influx [102]. Such a mechanism, however, could be blunted in the acidic PDAC microenvironment because of their inhibition by an intracellular acidification [103, 104]. It should be mentioned here that K_Ca_3.1 inhibition by an intracellular acidification was not seen in a recent study [105].

### 6.3 Tumor‐Infiltrating Immune Cells

The function of immune cells is strongly dependent on the ambient pH and lactate concentration. Both an increase in the proton or lactate concentration impairs immune cell function (see [106] for review). This is not only seen in solid tumors but also in lymph nodes. Paracortical zones of lymph nodes are markedly acidic with a pH_e_ as low as pH_e_6.3. This acidification is caused by lymphocyte metabolism [107]. The extracellular acidification impairs the secretion of many cytokines, which is an important effector function of lymphocytes [108]. When lymphocytes are activated by dendritic cells in an acidic environment (pH_e_6.6), their initial response is attenuated. However, when the same lymphocytes are then transferred to pH_e_7.4, their activation occurs normally. This pH_e_‐dependent behavior is seen as a safety measure to prevent premature activation of lymphocytes already within the lymph node. On the other hand, it was suggested that an alkalinization of tertiary lymphoid structures (TLS) might boost their role in antitumor immunity [107].

It is a consistent observation of the three in vivo studies cited in the preceding sections [36, 37, 74], that “pH targeting maneuvers” modify the immune cell infiltrate in PDAC. Notably, the antitumor effect of inhibiting NBCe1 (SLC4A4) is absent in immuno‐deficient mice, which clearly shows the importance of targeting pH regulation for tumor immunity. Ablation of NBCe1 in KPC pancreatic cancer cells resulted in a tenfold higher CD8^+^ T cell accumulation, increased CD8^+^/T_reg_ cell ratio, increased expression of the T cell activation marker CD69, and of IFNγ production. In sections of the tumor tissue, CD8^+^ T cells were found more frequently in the tumor center, and they were more efficient at killing pancreatic cancer cells devoid of NBCe1 [37]. It was claimed that an extracellular acidification made NBCe1‐deficient tumor cells more resistant against T cell‐mediated killing. However, unfortunately, these experiments were conducted in media whose pH was adjusted with the inaccurate “HCl titration method” [37]. Treating KPfC mice with the NHE1 blocker cariporide also leads to an altered immune cell infiltrate. The ratio of CD3^+^/Ly6G^+^ cells in tumor nodes rises in cariporide‐treated mice. Thus, NHE1 inhibition alters the immune cell infiltrate from a neutrophilic to a more lymphocytic one [36]. Treating KPCY mice with a CAIX inhibitor (SLC‐0111) diminishes the infiltration of B220^+^ B cells, while it does not affect CD3^+^ T cells [74].

So far, the underlying mechanisms by which “pH targeting maneuvers” impact the immune cell infiltrate in PDAC are not fully understood. Several possibilities could come into play. Due to the rigidity of their nucleus, lymphocytes may be hindered from migrating through the very dense mesh of the fibrotic PDAC microenvironment [109]. This deficit may be mitigated by the reduction of tumor fibrosis through NHE1 inhibition [36]. An extracellular acidification has long been known to produce anergy in tumor‐infiltrating CD8^+^ T lymphocytes, an effect that was reversed in vitro through normalization of the pH_e_ and in melanoma cell‐bearing mice by the application of the proton pump inhibitor esomeprazole [108]. T cell anergy could also be induced by the pH‐dependence of immune checkpoint regulators. Thus, VISTA (V‐domain immunoglobulin suppressor of T cell activation) suppresses T cells in an acidic environment such as that of the tumor microenvironment [110], and PD‐L1 expression is increased in breast cancer cells when they are cultured in an acidic medium [111].

Neutrophil behavior is also pH‐dependent. Their response to an extracellular acidification is at least partially mediated by an intracellular acidification. Chemotaxis in a C5a gradient is optimal at pH_e_7.0 and markedly impaired at pH_e_6.5 and in the presence of the NHE1 blocker cariporide. This is due to a pH_i_‐dependent inhibition of LTB_4_ secretion and Cdc42 activity [112]. On the other hand, neutrophil chemotaxis toward C5a is more efficient when the C5a gradient is paralleled by a gradient of the proton concentration [112]. When mice bearing orthotopically transplanted pancreatic cancer cells (KPCY2838) were treated with a C5a receptor blocker (CCX168), the immune cell infiltrate shifts from a neutrophilic to a lymphocytic one [113]. Thus, NHE1 activity, intratumoral proton and C5a gradients appear to collectively promote the accumulation of neutrophils (or myeloid‐derived suppressor cells) in the tumor stroma.

It remains to be determined whether other pH‐regulatory transport proteins such as H_V_1 proton channels also shape the immune cell infiltrate in PDAC. H_V_1 channels are functionally expressed in neutrophils [114] and polymorphonuclear myeloid‐derived suppressor cells isolated from subcutaneous Lewis lung carcinoma cell tumors [115]. It was speculated that they might sustain elevated NADPH‐oxidase activity in tumor‐associated macrophages or neutrophils. The resulting high concentration of reactive oxygen species would in turn dampen T cell function [116]. So far, it is not known whether somatic mutations H_V_1 found in multiple cancer types may affect cancer cell aggressiveness [117].

## 7 Conclusions

**FIGURE 2:**
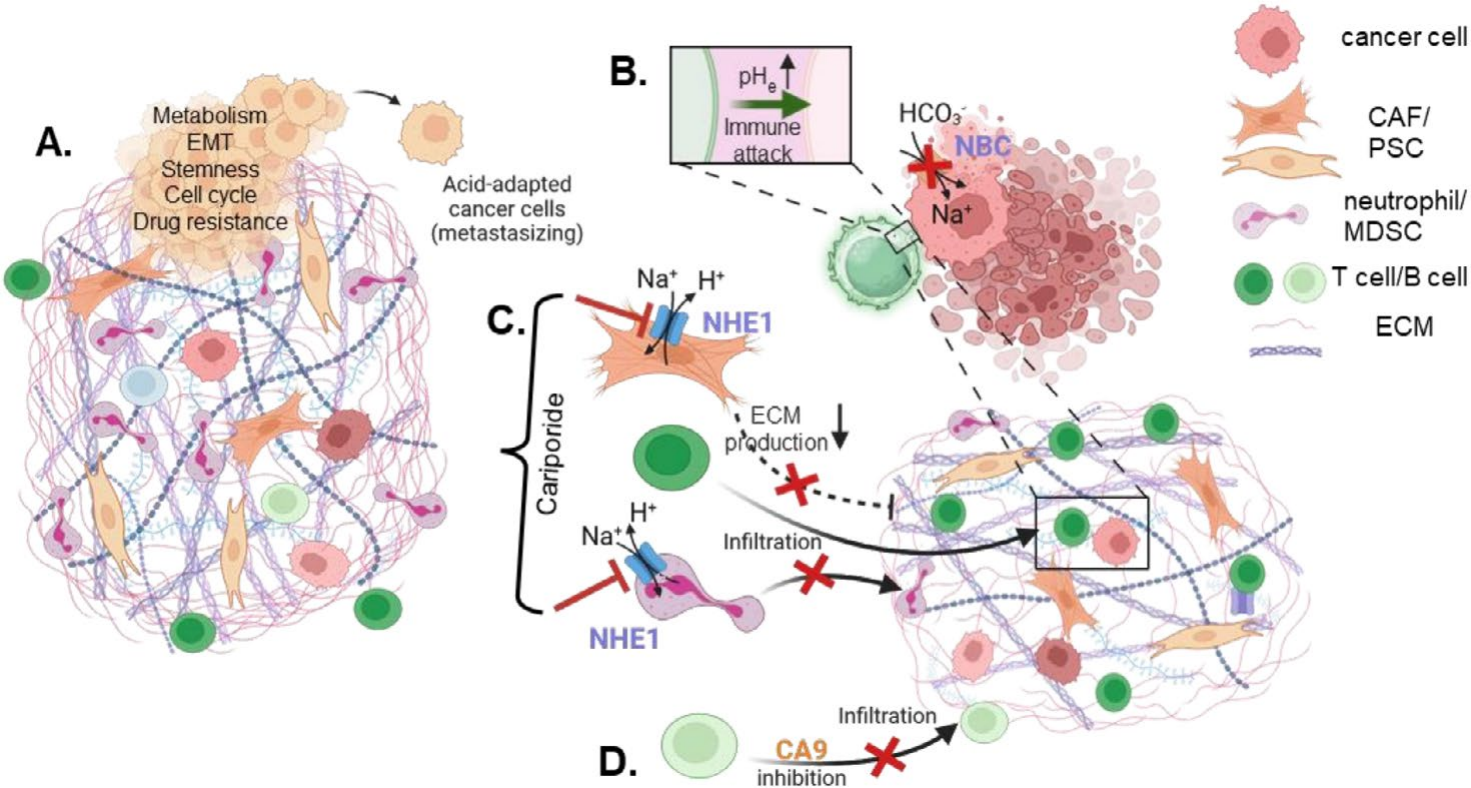
pH dynamics play a decisive role in PDAC pathophysiology and offer therapeutic opportunities. (A) Priming pancreatic cancer cells in the acidic tumor microenvironment profoundly alters their phenotype and promotes metastasis. (B–D) “pH‐targeting maneuvers” improve disease outcome in murine PDAC models. (B) Knockout or inhibition of NBCe1 (*Slc4a4*) improves tumor cell killing by activated T cells. (C.) NHE1 inhibition with cariporide reduces fibrosis and shifts the immune cell infiltrate from a neutrophilc to a lymphocytic one. (D) Combining gemcitabine with the carbonic anhydrase inhibitor reduces B cell infiltration into the tumor tissue. (This figure was created with Biorender.)

Recent years have seen a remarkable gain of knowledge on the role of pH in PDAC pathophysiology (see Figure 2). These new findings support the idea that the unique pH landscape found in the healthy pancreas and in PDAC contribute to disease progression. Having disentangled some of the underlying mechanisms, it becomes evident that “pH‐targeting maneuvers” may offer therapeutic potential. Here, the pH‐dependent modulation of antitumor immunity appears to stand out as a therapeutic opportunity whereby the impact on immune cells seems to be at least in part an indirect one: Tumor cell killing by immune cells depends on pH_e_ which, in turn, is regulated by the effect of pH‐regulatory transport proteins expressed in cancer cells (e.g., [37]). The inhibition or absence of pH‐regulatory transport proteins could lead to an increased vulnerablility of the cancer cells, or it could cause an altered pH‐nanoenvironment at the contact site between tumor cells and T cells. pH‐regulatory transport proteins such as NHE1 or H^+^/K^+^‐ATPase produce a distinct pH‐nanoenvironment at the cell surface [40, 77, 118]. The perimembranous pH_e_ nanoenvironment regulates integrin‐mediated focal adhesion dynamics in migrating cells [118] and formation of invadopodia [93, 119]. The formation of the immunological synapse also strongly depends on integrins [120]. Therefore, it would be intriguing to see whether high‐resolution imaging of pH dynamics at the immunological synapse reveals pH nanodomains that regulate integrin function and hence immuno‐signaling.

“pH‐targeting maneuvers” improve the efficacy of chemotherapy or immune checkpoint blockers. Therefore, the development of novel and highly specific inhibitors, for example, against NBCs, is urgently needed. Enormous progress made in obtaining structural information by cryo‐EM or by AI‐based analogy modeling opens a window of opportunity for accelerated drug discovery. It will be interesting to see whether these new drugs can be combined with antibody‐drug conjugates (ADCs), either by targeting transport proteins that are overexpressed in PDAC or in combination with other targets [121]. Antibodies, in turn, could be further modified by enabling them to recognize their target in a pH‐dependent manner [122, 123]. One could also increase the resilience and ability of CAR‐T cells to cope with the acidic tumor microenvironment by engineering them to express additional acid–base transporters. Innovative modalities to image the T cell response in PDAC [124] will offer the possibility to monitor the impact of the new drugs more directly. Taken together, the *pHioniC* project has laid a sound groundwork for future exciting developments.

## Author Contributions

**Albrecht Schwab:** conceptualization, writing – original draft, writing – review and editing. **Micol Rugi, Pawel Swietach, Wiktoria Błaszczak, Ivana Novak, Ganga Deshar, Stine Falsig Pedersen, Renata Ialchina, Albin Sandelin, Jiayi Yao, Stephan J. Reshkin, Rosa A. Cardone, Tiago M. A. Carvalho, Annarosa Arcangeli, Rayhana Bouazzi, Franco N. D'Alessandro, Natalia Prevarskaya, Madelaine M. Audero, Halima Ouadid‐Ahidouch, Julie Schnipper, Luis A. Pardo, Xiaoyi Shi, Frauke Alves, Jakub Mitręga, Anna Trauzold, Sofie E. Hagelund, György Panyi, Marco Cozzolino, Clemens M. W. G. Löwik, Laura Mezzanotte, Roisin McMorrow, Andreas Pahl, Torsten Hechler, Elena Papacharisi, Alessandra Fiorio Pla, Ildiko Szabo, Verena Hofschröer, Zoltán Pethő:** writing – review and editing.

## Funding

The authors were supported by the Marie Skłodowska‐Curie Actions Innovative Training Network (Grant 813834—*pHioniC—*H2020‐MSCA‐ITN‐2018). A.S and Z.P. received additional funding from Deutsche Forschungsgemeinschaft (GRK 2515 (Chembion) and PE 3917/2‐1).

## Ethics Statement

No ethics approval was required for this manuscript.

## Conflicts of Interest

The authors declare no conflicts of interest.

## Data Availability

The data that support the findings of this study are available on request from the corresponding author. The data are not publicly available due to privacy or ethical restrictions.
